# Pattern of Cerebral Palsy Among Sudanese Children Less Than 15 Years of Age

**DOI:** 10.7759/cureus.7232

**Published:** 2020-03-10

**Authors:** Karimeldin Salih

**Affiliations:** 1 Pediatrics, College of Medicine, University of Bisha, Bisha, SAU

**Keywords:** cerebral palsy, quadriplegia, spastic, antenatal, postnatal, socioeconomic

## Abstract

Background: Cerebral palsy (CP) is a non-progressive, everlasting neurological disorder of movement, posture, and physical activities, with a prevalence of 2.2-3.3/1,000. CP is a condition that occurs globally, with a similar prevalence in both developed and undeveloped countries. However, the etiology differs according to the socioeconomic status of the countries. The objective is to determine the pattern and the contributing factors of CP among Sudanese children.

Methods: This was a retrospective hospital-based study conducted over a period of three years in a pediatric referral hospital in Khartoum, Sudan. One hundred and eight patients of CP were enrolled, of whom 59 (54.6%) were males and 49 (45.4%) were females.

Results: Spastic quadriplegic CP was the most common type. Most cases were from lower social classes. Prenatal, antenatal, and unclassified CP were found in 45 (41.7%), 31 (28.7.%), 23 (21.3%), and 9 (8.3%) cases, respectively. Birth asphyxia, neonatal jaundice, Toxoplasma gondii, rubella virus, cytomegalovirus, herpes simplex virus infections (TORCH), and sepsis (acquired) were the main causative factors.

Conclusion: Spastic quadriplegia is the most common type of CP. Most of the cases had a direct positive relationship with socioeconomic status. The prenatal period was the most common period for the development of CP.

## Introduction

Different patterns of neurological diseases have been observed in different settings. The most common disorders are epilepsy, cerebral palsy (CP), post-febrile seizures, and auditory and communication disorders [1]. Studies on CP patterns in relation to birth weight showed that infants of very low birth weight (VLBW) i.e., less than 1,500 g are 20 to 80 times more likely to develop CP than infants of birth weight of more than 2,500 g [2]. CP is a non-progressive, everlasting disorder of movement, posture, and physical activities, with a prevalence of 2.2-3.3/1,000 in children from developing countries. However, the data in Africa is not well-documented [3]. No published data from Sudan reflects the magnitude of CP and neurological disorders and disabilities [4].

CP is a condition that occurs globally, with a similar ratio among both developed and undeveloped countries. However, the etiology differs according to the socioeconomic status of the countries [5]. Congenital hemiplegia is the most common form of CP among children born at term and second to diplegia among children born prematurely [6]. Antenatal causes contribute largely to CP in developed countries, while secondary causes like infection, asphyxia, and jaundice are more commonly found in developing countries [7]. Although the exact etiology of CP is not fully defined, the insult to the developing brain, chromosomal abnormalities, congenital infection, or difficult labor can contribute to permanent and non-progressive disorders, which manifest early in life [8]. CP may cause a range of associated problems, including hearing and visual deficits, nutritional and feeding problems, respiratory infections, epilepsy, and cognitive and communicative impairments in children [9].

The predictors of CP must be determined to generate basic data to produce interventional measures to reduce the risk of CP. A study of possible risk factors to CP will alert health authorities and health planners to work hard to minimize the serious effects of CP [10]. The lack of equipment, instruments, and manpower needed for the follow-up of pregnant ladies and their neonates thereafter might explain the magnitude of CP in developing countries, including Sudan [11]. The ultimate complications might include mental impairment, physical disabilities, visual and hearing problems, as well as speech difficulties [12]. Complications in the perinatal period alert for comprehensive emergency obstetric and neonatal care. Children over five years of age, children with severe CP, and children with intellectual disabilities were at greater risk of malnutrition. The approach to the assessment and management of children and youth with CP depends, to a considerable extent, on the frameworks used to conceptualize diseases and disorders. The recent publication of the World Health Organization (WHO) International Classification of Function, Health, and Disability (ICF) provides an opportunity to integrate several perspectives about this important and prevalent group of childhood disabilities [13]. This study was conducted to determine the pattern and contributing factors of CP among Sudanese children.

## Materials and methods

Study design

This was a retrospective hospital-based study conducted over three years in a pediatric referral hospital in Khartoum, Sudan. One hundred and eight CP patients were enrolled, 59 (54.6%) males and 49 (45.4%) females. The ethical committee of the pediatric hospital in Khartoum approved the study. Consent was taken from the parents/guardians.

Procedures and process

The patients were consulted and examined in the outpatient department (OPD). A questionnaire was designed by experts in pediatrics to get information from the patients or caregivers regarding names, gender, socioeconomic status of parents, pregnancy, delivery, neonatal history, and developmental milestones. A thorough general examination in general and central nervous system examination, in particular, was done for each patient by the author, who is a pediatrician. The Surveillance of Cerebral Palsy in Europe (SCPE) classification was used in this study to classify types of CP [14]. A modified version of Kuppuswamy’s socioeconomic scale was implemented to determine the patients’ socioeconomic status [15]. A pilot study was conducted first in 20 cases.

Inclusion and exclusion criterion

Diagnosed cases as CP by history, examination, computed tomography (CT), magnetic resonance imaging (MRI), and confirmed diagnosis by two pediatricians were included. Unconfirmed CP among patients aged less than one year was excluded.

Statistical analysis

Statistical Package for the Social Sciences; version 21 (SPSS Inc., Chicago, IL) description statistic was used; P value less than 0.05 was considered significant.

## Results

Clinical patterns of CP

The total number of children with CP included in this analysis was 108. The males numbered 59 (54.6%), and the females numbered 49 (45.4%). The difference in frequency was insignificant (P > 0.05). The most common types of CP were spastic quadriplegic, spastic hemiplegic, spastic diplegia, hypotonic, ataxic, mixed, and unclassified. CP was found more frequently among the lower social class (Table 1).

The most common type of CP in children is spastic quadriplegic (43.5%), with a significant association between it and social classes (p < 0.05). Meanwhile, no significant difference was found with regard to gender (p > 0.05). The frequencies of spastic quadriplegic, spastic hemiplegic, spastic diplegia, hypotonic, ataxic, mixed, and unclassified CP were 47 (43.5%), 28 (25.9%), 15 (13.9%), 9 (8.3%), 4 (3.7%), 3 (2.8%), and 2 (1.9%), respectively (Table 1).

The most common etiology was perinatal (41.7%) from birth asphyxia (26.9%) and neonatal jaundice (14.8%). CP was a consequence of perinatal, antenatal, acquired, and unclassified causes in 45 (41.7%), 31 (28.7%), 23 (21.3%), and 9 (8.3%) patients, respectively. Twenty-nine (26.9%) cases were due to birth asphyxia, 16 (14.8%) were attributed to neonatal jaundice, 31 (28.7%) were due to TORCH (as antenatal), and only 23 (21.3%) had sepsis (as acquired) (Table 2). Nine children with CP (8.3%) had unclassified causes. Seventy (64.8%), 17 (15.7%), 11 (10.2%), 6 (5.6%), and 4 (3.7%) children with CP belonged to the lower, upper-lower, lower-middle, upper-middle, and upper social classes, respectively (Table 1).

**Table 1:** Time and Type of Brain Insult for Cerebral Palsy Patient

Time and Type of Brain Insult for Cerebral Palsy Patient			
Time of the Problem	No. of Cases	Frequent Pattern of Insult	Main Type of Insult
Perinatal	45	29 (26.9%)	Birth asphyxia
		16 (14.8%)	Neonatal jaundice
Antenatal	31	28.7%	TORCH
Acquired	23	21.3%	Sepsis
Undetermined	9	8.3%	Unknown causes
Total	108		
The most common etiological insult causes were birth asphyxia and sepsis.			

**Table 2:** Patterns of Cerebral Palsy among Sudanese Children

Patterns of Cerebral Palsy among Sudanese Children								
Type of Cerebral Palsy	Gender		Social Class					Total
	Male	Female	Upper	Upper-middle	Lower-middle	Upper-lower	Lower	
Spastic quadriplegic	26	21	2	1	6	7	31	47 (43.5%)
Spastic hemiplegic	13	15	1	4	0	7	16	28 (25.9%)
Spastic diplegia	8	7	0	1	4	1	9	15 (13.9%)
Hypotonic	5	4	0	0	0	2	7	9 (8.3%)
Ataxic	3	1	1	0	1	0	2	4 (3.7%)
Mixed	2	1	0	0	0	0	3	3 (2.8%)
Unclassified	2	0	0	0	0	0	2	2 (1.9%)
Total	59 (54.6%)	49 (45.4%)	4 (3.7%)	6 (5.6%)	11 (10.2%)	17 (15.7%)	70 (64.8%)	108 (100%)
Spastic quadriplegic is the most common CP type. The most affected group in the community are those from lower social class								

## Discussion

CP has been described as “a group of non-progressive but often changing motor impairment syndromes secondary to lesions or anomalies of the brain arising in the early stages of development” [16]. Males are affected more than females, as proven by studies done in Africa [16]. The reason for this is still not well investigated; however, other studies have found equal gender distribution [17]. Globally, the pattern of CP prevalence per 1,000 neonatal survivors in the higher birthweight group is similar to that seen in the prevalence per 1,000 live births. By contrast, the pattern per 1,000 neonatal survivors in the group of birth weight of less than 1,000 g differs from that seen in live-birth prevalence [18].

The most common types of CP among Sudanese children are spastic quadriplegia, spastic hemiplegia, hypotonic, ataxic, and mixed, a finding that is in agreement with a study done locally in Khartoum, Sudan, and in South Africa and other African countries [8,19]. However, little is known about the prevalence, incidence, and guidelines for CP management in low-resource countries such as African countries. Moreover, several studies have identified barriers toward optimal care for children with CP in Africa. Limited access to health-care facilities and specialists, as well as a lack of adaptive equipment such as wheelchairs and other ambulation aids, contribute to the treatment gap for children with CP [20].

Meanwhile, in Sudan, the majority of the patients, 87 (80%), originated from the lower class, a finding aligned with a study done among other African countries [20]. Such a case is not unusual for developing countries, where infections are common, facilities for neonatal care are lacking, inappropriate investigation tools are used, and a lack of proper mother and child health care is observed, which makes infection the top problem leading to CP [21,22]. Poverty is the underlying cause of insufficient nutrition and has an impact on health facilities’ implementation besides the high prevalence of CP among children [23]. Most cases of CP occurred prenatally (41.7%), followed by antenatal (28.7%) and acquired causes 23 (21.3%). Recent clinical patterns of CP have shown an increase in lower spastic quadriplegic cases (28.7%) among Sudanese children. This agrees with other authors who have reported that several risk factors may increase the likelihood of a developmental brain injury [24,25]. Poor maternal health and low birth weight are just some of the risk factors for any type of CP. The lower social class was found to be the most common social class for the development of CP, which is in harmony with other studies in Africa; the time of brain insult during prenatal, antenatal, and acquired causes agrees with other studies [26-28]. Birth asphyxia was presented as the main etiological cause of CP that occurs prenatally, followed by neonatal jaundice; this is in harmony with a study done in Nigeria [29].

This article is aimed to alert policy-makers to address issues regarding pregnancy, delivery and neonatal management. The limitation of the study was the amount of data gathered; more data is required to validate the findings. The study also recommends meticulous care regarding mother and child care.

## Conclusions

CP which is a non-progressive disorder affecting movement, posture and child activity, is one of the common neurological disorders among children. Diagnosed cases through clinical examination and investigation were enrolled in this study. Patient personal data were collected from care givers, followed by general and focused examination by the authors. Males developed CP more than females; spastic quadriplegia is the most common type of CP, as proven by other national and international studies. Most of the cases have a positive relationship with socioeconomic status, which is usually low in developing countries, and the prenatal period presents the most common period for CP development. The most common etiology was perinatal. This study draws the attention of the policy makers to anticipate and prevent possible precipitant factors such as socioeconomic status for the emergence of CP.
